# Effect of POSS Particles and Synergism Action of POSS and Poly-(Melamine Phosphate) on the Thermal Properties and Flame Retardance of Silicone Rubber Composites

**DOI:** 10.3390/ma11081298

**Published:** 2018-07-27

**Authors:** Przemysław Rybiński, Bartłomiej Syrek, Dariusz Bradło, Witold Żukowski

**Affiliations:** 1 Faculty of Mathematics and Natural Science, Jan Kochanowski University, Świętokrzyska 15, Kielce 25-406, Poland; bartlomiejsyrek@wp.pl; 2 Faculty of Chemical Engineering and Technology, Cracow University of Technology, Warszawska 24, Cracow 31-155, Poland; dariusz.bradlo@gmail.com (D.B.); pczukows@gmail.com (W.Ż.)

**Keywords:** silicone rubber, polyhedral oligomeric silsesquioxanes (POSS), melamine polyphosphate, thermal properties, flammability

## Abstract

This article presents flame retardant compounds for silicone rubber (SR) in the form of polyhedral oligomeric silsequioxanes (POSS), containing both isobutyl groups and amino-propyl (AM-POSS) or chloro-propyl group (HA-POSS) or vinyl groups (OL-POSS). Silsequioxanes were incorporated into the silicone rubber matrix in a quantity of 3 and 6 parts by wt by the method of reactive stirring with the use of a laboratory mixing mill. Based on the analyses performed by TG (Thermogravimetry) FTIR (Fourier Transform Infrared Spectroscopy), conical calorimeter, and SEM-EDX (Scanning Electron Microscopy and Energy Dispersive X-ray Spectroscopy) methods, the thermal degradation mechanism of non-cross-linked and cross-linked silicone rubber has been elucidated. The effects of POSS, and POSS in a synergic system with melamine polyphosphate (MPP), on the thermal properties and flammability of silicone rubber composites were presented. Based on the test results obtained, a mechanism of flame retardant action POSS and POSS-MPP has been proposed. It has been shown that POSS, especially with MPP, considerably increases the thermal stability and decreases the flammability of the SR rubber composites under investigation.

## 1. Introduction

Silicone rubbers (SR) are characterized by excellent compatibility, and chemical, thermal, and UV resistances, and also showing very good dielectric properties. Unfortunately, silicone rubbers are flammable polymers; when ignited they are totally burnt, releasing great amounts of heat within a short period of time. The flammability of silicone rubbers considerably reduces their use in the medical, automotive, power, and cable industries [1,2].

A commonly used method of improving the resistance of polymeric materials, including silicone rubbers, against the action of fire consists of incorporating additive flame-resistant compounds into their matrix. On account of environmental restrictions concerning the use of halogen flame retardants, at present halogen-free organophosphorus flame-retardant compounds, such as melamine polyphosphate (MPP) or ammonium polyphosphate (APP), have the greatest application potential [3].

The action of organophosphorus compounds consists in forming an expanded, intumescent carbon layer on the polymer surface during its thermal decomposition. This layer acts as a barrier preventing heat transfer from its source to the polymer surface and reducing the fuel transfer to the flame [4].

To obtain a high efficiency of flame retardant compounds, one has to incorporate them into the polymeric matrix in the presence of carbon-rich compounds.

During the thermal decomposition of a polymeric composite, the organophosphorus compounds contained in it, e.g., MPP, undergo degradation to water, ammonia, and PP acid that when reacting with carbon compounds, forms thermally unstable esters of phosphoric acid. Their decomposition, mainly by dehydration processes, results in the formation of a carbon layer cutting off the flow of oxygen, fuel, and heat between the polymer and flame. Water vapor and nitrogen compounds formed during the MPP decomposition diffusing to the flame, foam the liquid destruction products of the composite and increase the insulating power of the boundary layer [5].

The incorporation of MPP into the poor in carbon silicone rubber to make it flame retardant must be combined with a carbonizing additive, such as pentaerythritol (PER) [6].

From the investigations performed so far, it distinctly follows that the satisfactory effectiveness of the MPP/PER system, especially in relation to silicone rubber, can be obtained when its content is over 30 parts by wt. Unfortunately, such a high quantity of this flame retardant negatively affects the processing, functional properties, and price of the silicone rubber composites obtained [7].

New, alternative of halogen and halogen-free flame-retardant compounds are polyhedral oligomeric silsequioxanes (POSS) [8,9]. POSS have the structure of cage whose edges are built of combined silicone and oxygen atoms. Organic ligands are directly linked to silicon atoms, owing to which, the cage can be combined with polymer chains by means of covalent bonds, forming hybrid composites. The size of silicate cages ranges from 1 to 3 nm, hence they are treated as the smallest silica particles to be obtained [10].

POSS can be incorporated as reactive components during polymerization or combined with macromolecules by their direct plasticization with the polymeric matrix.

From the investigations performed so far, it follows that POSS incorporated into silicone rubbers considerably improves their mechanical and thermal properties and reduces their flammability [11]. Liu et al. [12] have found that POSS with various functional groups, such as phenyl-trisilanol, isobutylo-trisilanol, and isobutyl-monosilanol groups, increases the thermal stability of silicone rubber nanocomposites. As a result of the tests performed, it has been shown that room temperature vulcanized silicone rubber (RTV-SR), containing 5% of tri-silanol-isobutyl POSS, is characterized by the highest thermal stability among the composites tested. Other studies have shown a synergic effect of silica and POSS on the improvement in thermal and mechanical properties of silicone rubber (RTV-SR) [12].

The use of POSS as effective compounds for reducing the flammability of polymers is presented in a review by Zhang and co-workers [9]. From the review of the source literature, it follows that in the past, attempts were undertaken to examine and explain the mechanism of the synergic action of the POSS-phosphorus compounds on the flammability of polypropylene, polylactide, polyester, and epoxide resins or butyl polysuccinate [11,13,14,15,16,17,18].

Carosio et al. [19] studied the effect of layer by layer assemblies on the thermal and combustion behavior of acrylic fabrics. Bi-layers consisting of APP/POSS pairs have been deposited on acrylic fibers, creating a homogeneous and regular coating able to completely cover the fibers.

Researchers indicated that the thermal degradation in an inert atmosphere, as well as the thermo-oxidation in air of the acrylic fibers, has been significantly modified by the coatings due to their char-former character [19].

Despite the investigations performed, the effect of POSS, especially the POSS-phosphorus compounds system, on the flammability of polymeric materials has not been totally explained.

The present paper describes pioneering studies on the effect of three POSS compounds containing amino groups (AM-POSS), chlorine groups (HA-POSS), and vinyl groups (OL-POSS) on the thermal properties and flammability of high-temperature vulcanized silicone rubber (SR), also taking into account the decomposition kinetics. The effect of the synergism of POSS-MPP action on the flammability of silicone rubber was also examined. Based on the analyses performed by TG, FTIR, conical calorimeter and SEM-EDX methods, the mechanism of the flame-retardant action of POSS-MPP was explained.

## 2. Experimental

### 2.1. Materials

The object of study was methylvinylsilicone rubber (SR), made by “Silikony Polskie” (Nowa, Sarzyna, Poland), with a molecular weight of 60–70 × 10^4^ (kg/mol) and vinyl group content of 0.05–0.09%. The rubber was cross-linked with the use of bis(2,4-dichlorobenzoil) peroxide from “Silikony Polskie” in a quantity of 0.7 part by wt/100 parts by wt. of the rubber (Vulcanised silicone rubber signed as SR OP, where OP is organic perioxide).

**Scheme 1:**
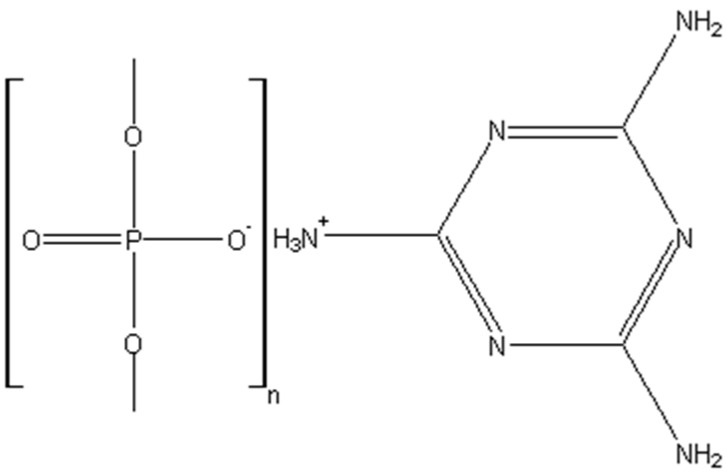
Melamine polyphosphate structure.

The following compounds were used as fillers for the rubber blends: melamine polyphosphate MPP from Everkem (Milano, Italy) (Scheme 1), chemical formula of MPP is C_3_H_6_N_6_ (H_3_PO_4_)_n_, and polyhedral oligomeric silsesquioxanes POSS from Hybridplastics (Hattiesburg, USA). The chemical formula of POSS is (RSiO_1.5_)_n_ where R is a hydrogen atom or an organic group (Table 1). Please give the manufacture’s location.

### 2.2. Methods

#### Preparation of Rubber Blends

Rubber blends were prepared using a laboratory two-roll mill, with roll dimensions of *D* = 140 mm and *L* = 300 mm. The average temperature of the rolls was 35 °C. The rotational speed of the front roll was *V*p = 20 rpm, friction 1:1.

The composition of the rubber mixtures are shown in Table 2.

Kinetic parameters of vulcanization of the elastomer blends were measured by means of MonTech DRPA 300 Rheometer (Buchen, Germany), at 160 °C, according to standard PN-ISO 3417:1994, directly after the preparation of the rubber blends, and for comparison, after a week of conditioning at the ambient temperature. The blends were vulcanized in steel molds placed between electrically heated press shelves.

### 2.3. Experimental Techniques

#### 2.3.1. FTIR Analysis

Fourier transform infrared (FTIR) spectra were recorded on a FTIR Spectrum Two spectrophotometer from PerkinElmer (Waltham, MA, USA). Attenuated total reflection (ATR) accessory equipped with a single reflection diamond ATR crystal on ZeSe plate was used for all of the analysis. Measurement parameters were 32 scans from 500–4000 cm^−1^ in transmittance mode with a resolution of 8 cm^−1^ and the speed of scanning equal to 0.6329 cm·s^−1^.

#### 2.3.2. EDS Techniques

EDS analysis of residue after burning were obtained with the use of scanning electron microscope (SEM), model TM3000 from Hitachi (Krefeld, Germany).

#### 2.3.3. Thermal Properties

The thermal properties of the rubber composites were tested under an air atmosphere at a temperature ranging from 25 to 700 °C with the use of a thermal analyser (Jupiter STA 449F3, Netzsch Company, Selb, Germany). Weighed portions amounted to about 5–10 mg. Investigated samples were analyzed at a heating rate of 10 °C/min.

Determination of activation energy of destruction studied silicone rubber compositions.

In order to determine the activation energy of destruction, compositions of silicone rubber were tested under a nitrogen atmosphere at temperatures ranging from 25 to 700 °C with the use of a thermal analyser (Jupiter STA 449F3, Netzsch Company, Selb, Germany). The samples were heated with the rate 5, 10, 15, and 20 °C/min.

In general, the thermal degradation reaction of a solid polymer can be simply shown as: Bsolid → Csolid + Dgas, where Bsolid is the starting material, and Dgas and Csolid are the different products during the degradation of Bsolid. In thermogravimetric measurements, the degree of decomposition (conversion) can be calculated as follows [20]:

(1) $$X=\frac{W_{0}-W_{t}}{W_{0}-W_{f}}\;$$

where *X* is degree of decomposition; *W_t_*, *W*_0_, *W_f_* are the actual, initial, and final mass of the sample, respectively. A typical model for the kinetic process can be expressed as:

(2) $$\frac{dX}{dt}=kf(X)\;$$

where *dx/dt* is the decomposition rate, *f(X)*, the function of *X*, which depends on the particular decomposition mechanism, and *k* is the decomposition rate constant, which can be expressed by the Arrhenius equation:

(3) $$k=A\exp\left(\frac{-E}{RT}\right)\;$$

where *A* is the pre-exponential factor (s^−1^), *E* is the activation energy (J/mol), *R* is the gas constant (8.314 J·mol^−1^·K^−1^), and *T* is the temperature (K). Substituting Equation (3) into Equation (2), gives

(4) $$\frac{dX}{dt}=A\exp\left(\frac{-E}{RT}\right)f\left(X\right)\;$$

If the temperature of a sample is changed by a constant value of *β* (*β* = *dT/dt*), the variation of the degree of decomposition can be analyzed as a function of temperature. Therefore, the reaction rate gives:

(5) $$\frac{dX}{dT}=\frac{A}{\beta }\exp\left(\frac{-E}{RT}\right)f\left(X\right)\;$$

Equations (4) and (5) are the basic equations for the kinetic calculation.

This iso-conversional integral method, suggested independently by Flynn and Wall and Ozawa [21,22], uses Doyle’s approximation of the temperature integral. This method is based on the equations:

(6) $$\log\beta =\log\frac{AE}{Rg(\alpha )}-2.315-\frac{0.457E}{RT}\;$$

where decomposition in kJ/mol; *α*, the degree of conversion; *T*, the absolute temperature to reach the conversion, and is the integral conversion function

(7) $$g(\alpha )=\int_{0}^{\alpha }\frac{d\alpha }{f(\alpha )}\;$$

is the integral conversion function

Thus, at a constant conversion (*α* = const.), the plot log *β* versus (1/*T*), obtained from a series experiments performed at several heating rates, should be a straight line whose slope allows for the evaluation of the activation energy:

(8) $$slope=\frac{d(\log\beta )}{d(1/T)}=0.4567\left(\frac{E}{R}\right)\;$$

To apply this iso-conversional method, heating rates of 5, 10, 15, and 20 °C/min were chosen. In this study, the conversion values of 5, 15, 20, 25, 30, 35, 40, 45, 50, 55, 60, 65, and 70% have been used, which would give α values of 0.05, 0.15, 0.2, 0.25, 0.3, 0.35, 0.4, 0.45, 0.5, 0.55, 0.6, 0.65, and 0.7 respectively for the Flynn-Wall-Ozawa method.

#### 2.3.4. Flammability

The SR composites were examined using a cone calorimeter from Fire Testing Technology Ltd. (East Grinstead, UK). Elastomer samples with dimensions of (100 × 100 ± 1) mm and thickness of (2 ± 0.5) mm were tested in a horizontal position with a heat radiant flux density of 35 kW·m^−2^. During the tests, the following parameters were recorded: initial sample weight, time to ignition (TTI), sample weight during testing, total heat released (THR), effective combustion heat (EHC), average weight loss rate (MLR), heat release rate (HRR), and final sample weight.

## 3. Results

### 3.1. Thermal Properties of Uncured and Cured Silicone Rubber

The thermal degradation of polysiloxanes (silicone rubbers) can proceed according to three reaction mechanisms: unzipping mechanism, random scission of Si–O bond in the main chain of polymer, and external catalytic mechanism.

**Scheme 2:**
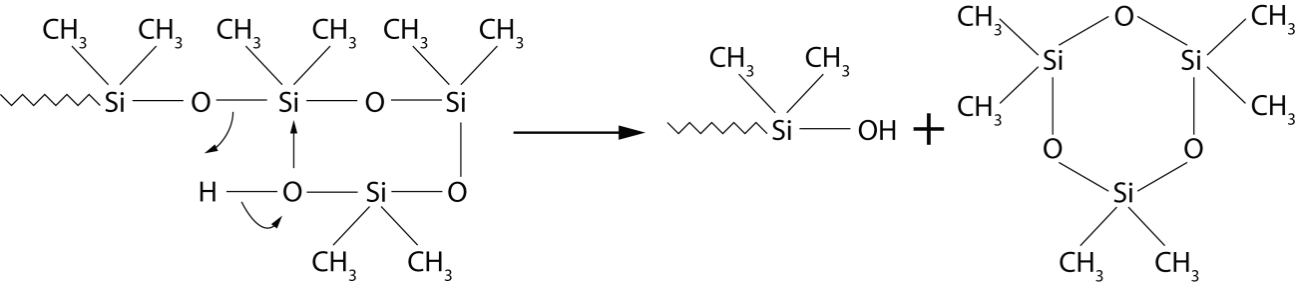
Inter-molecular mechanism degradation of hydroxyl-terminated polysiloxane.

Polydimethylsiloxanes (PDMS), containing silanol (Si–OH) or hydroxyl-alkyl-silanol (Si–R–OH) terminal groups, undergo thermal degradation according to the unzipping mechanism (Scheme 2) [20,23,24].

In the first stage of heating, the molecular weight of the polymer violently increases as a result of intermolecular condensation processes proceeding between terminal silanol groups and inter-chain Si–O groups. Further increase in temperature causes a linear decrease in the polymer molecular weight as a result of the formation of cyclic siloxanes followed by their tearing off and passing to the gas phase of cyclic siloxanes, mainly trimers and tetramers. Presumably, the cyclic siloxane that is stable at the polysiloxane degradation temperature can catalyze the resultant degradation processes of polysiloxane.

The weakest bond in the structure of polydimethylsiloxane (PDMS) is the C–Si bond, whose energy amounts to 326 kJ/mol. Nevertheless, cyclic siloxanes are formed by the decomposition of a Si–O bond, whose energy value is 451 kJ/mol, which in turn suggests that the thermal decomposition of PDMS should be considered from the point of view of both kinetic parameters and molecular structure and not only the bond energy.

**Scheme 3:**
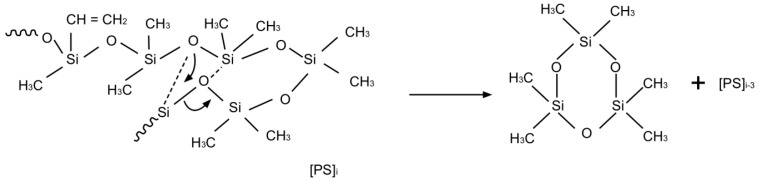
Mechanism degradation of polysiloxsane by random scission of Si–O bond.

Polysiloxanes containing terminal groups, such as a trimethylsilyl or vinyl group, as well as accidently occurring vinyl groups in the main chain, undergo thermal degradation by inter- or intra-molecular reactions proceeding between accidental bonds of the main polymer chains (Scheme 3) [23,25].

Unlike the unzipping mechanism, the thermal degradation of polysiloxanes according to the random scission mechanism requires decisively higher temperatures, which causes a violent loss of the sample mass, which is observed already in the early stage of the thermal decomposition of polysiloxane, while the scission of the Si–O bond resulting in the formation of oligomeric final products generally occurs in the middle part of polysiloxane chain.

Polysiloxanes containing ionic polar additives or impurities undergo thermal degradation according to a catalytic mechanism. Contrary to the unzipping and random scission mechanisms, the catalytic mechanism consists in hydrolytic decomposing the Si–O bond in the main polymer chain. Grassie and Macfarlane have shown that even trace quantities of KOH dramatically deteriorate the thermal stability of polysiloxanes, even within the range of moderate temperatures [26].

Radhakrishnan [24], investigating the processes of thermal decomposition of polysiloxanes terminated with vinyl group and hydroxyl group, has shown that PDMS terminated with vinyl group is thermally decomposed according to the random scission mechanism, while in the case of PDMS containing hydroxyl terminal groups, a mixed mechanism is noticeable, which means that this polymer is decomposed with the use of terminal hydroxyl groups, according to the unzipping mechanism and the random scission mechanism.

**Figure 1:**
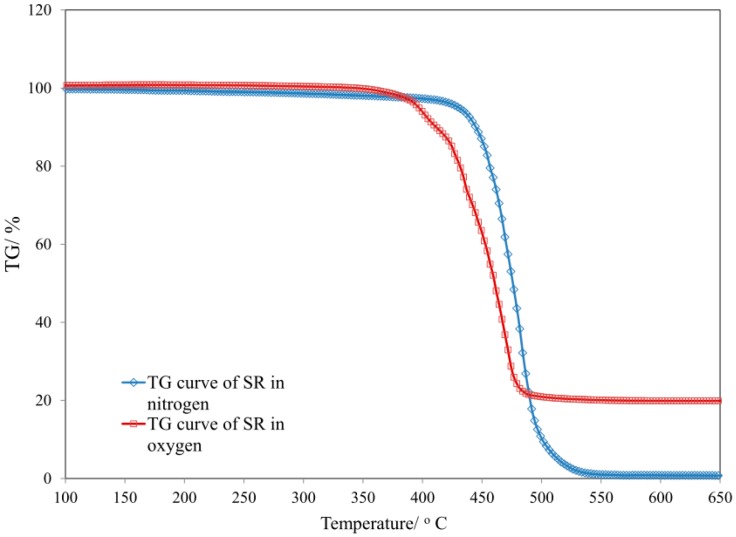
TG curves of silicone rubber in the air and nitrogen atmosphere.

**Figure 2:**
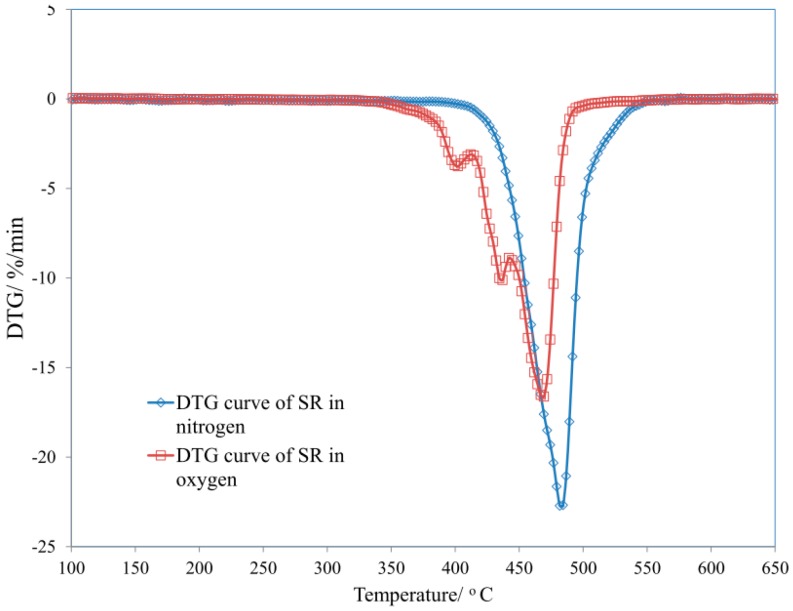
DTG curves of silicone rubber in the air and nitrogen atmosphere.

The methylvinylsilicone rubber tested in the atmosphere of a nitrogen gas underwent thermal decomposition in a single stage. The maximal rate of this process at a temperature of 483 °C amounted to 22.85%/min (Figure 1 and Figure 2). Under air, this rubber was thermally decomposed in three stages (Figure 1 and Figure 2). The first stage began at *T* = 350 °C, while the maximal rate of its decomposition at *T* = 396 °C amounted to 3.80%/min, the second stage began at *T* = 410 °C, while its maximal rate at *T* = 465 °C amounted to 16.90%/min (third stage).

The three-stage decomposition of silicone rubber under air can result from the catalytic action of air in the depolymerization reactions of polysiloxane to volatile cyclic low-molecular compounds, as confirmed by a decisively lower temperature of the beginning of thermal decomposition, TR, of SR rubber under air in relation to the value of TR under neutral gas. The cyclic compounds, formed as a result of the decomposition of SR rubber according to the random scission mechanism, are then thermally decomposed under both air and nitrogen at a temperature above 450 °C.

**Scheme 4:**
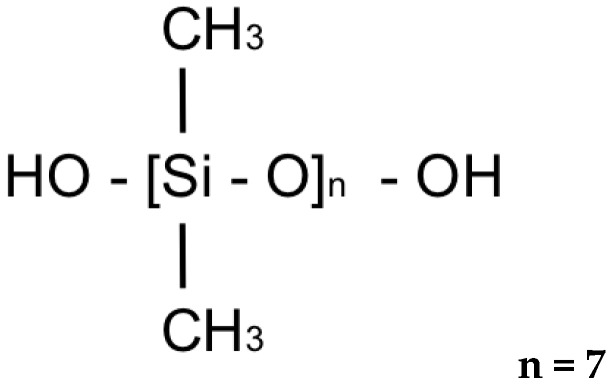
Structure of low molecular plasticizer.

The cross-linking of the methylvinylsilicone rubber decisively changes the character of its thermal decomposition under air. A small weight loss at Δ*T* between 60 and 395 °C is connected with the presence of plasticizer in the form of low-molecular hydroxydimethylsiloxane, incorporated into the elastomer blend (Scheme 4).

The temperature of the beginning of thermal decomposition of cross-linked silicone rubber was increased by 50 °C in relation to the non-cross-linked rubber. Also, the temperature of the maximal decomposition rate of the cross-linked rubber, amounting to *T*_RMAX_ = 505 °C, was increased by 45 °C in relation to the non-cross-linked rubber.

**Figure 3:**
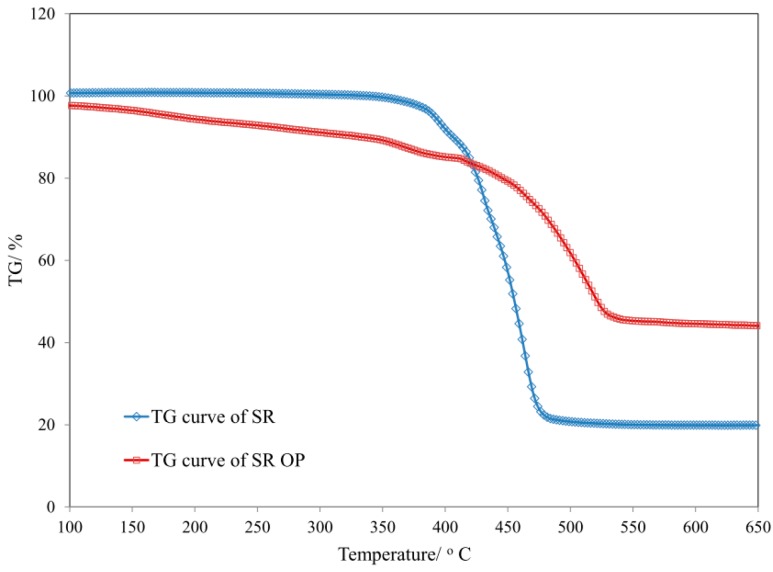
TG curves of uncured (SR) and cured (SR OP) silicone rubber.

**Figure 4:**
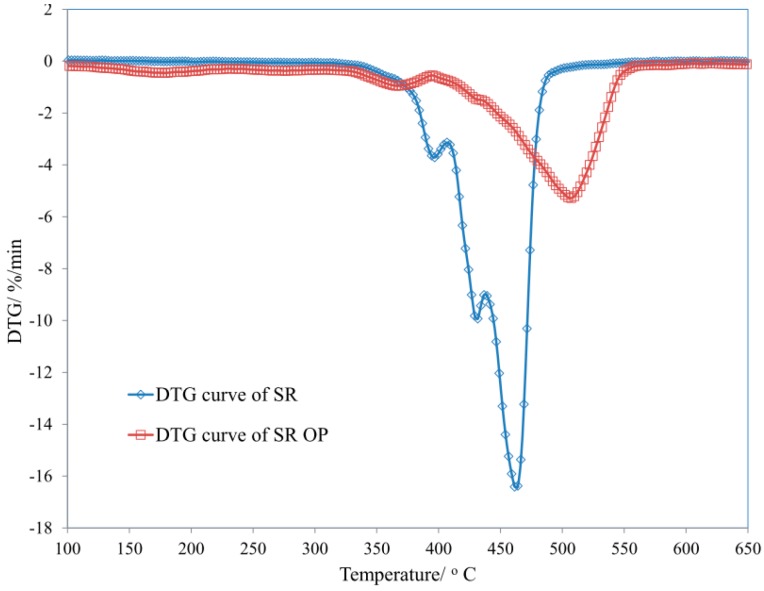
DTG curves of uncured (SR) and cured (SR OP) silicone rubber.

The cross-linking process of SR rubber also caused a considerable reduction in its thermal decomposition rate. Parameter *dm*/*dt* of non-cross-linked rubber amounts to 16.5%/min, while that of cross-linked rubber is 5.25%/min. The thermal decomposition residue was also considerably increased, amounting to 5.25% for the non-cross-linked rubber and 19.95% for the cross-linked rubber Figure 3 and Figure 4.

The temperature increase in the decomposition of cross-linked silicone rubber in relation to that of non-cross-linked rubber caused a radical mechanism to predominate in the decomposition of the cross-linked polymethylvinylsiloxane. An increase in temperature caused a hemolytic dissociation of the Si–C bond, which resulted in the formation of macro-radicals whose recombination and cross-linking decrease the elasticity of the polysiloxane chain and consequently impeded further formation of cyclic oligomers.

### 3.2. POSS Characteristic

**Figure 5:**
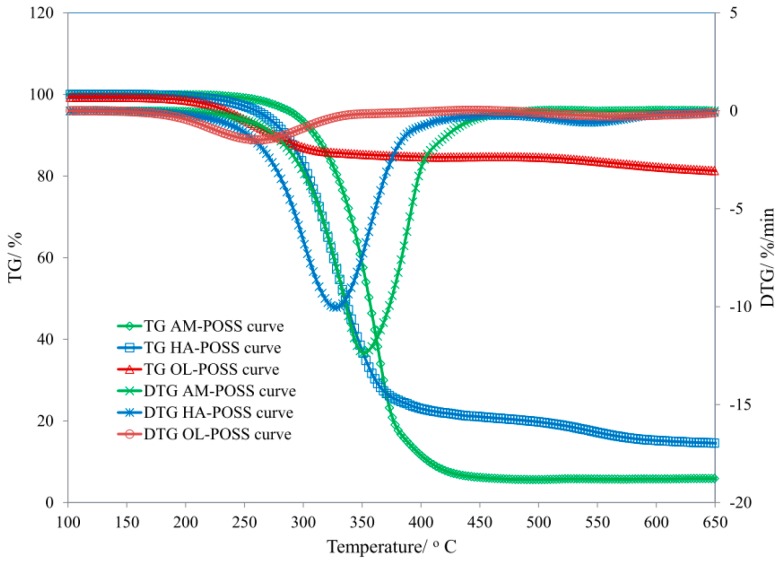
Thermal curves of POSS.

Figure 5 presents TG and DTG curves of the POSS under investigation. Aminopropylisobutyl silsequioxane (AM-POSS) underwent thermal decomposition in a single stage that began at *T* = 250 °C. The maximal rate of this process at T_RMAX_ = 365 °C amounted to 16.95%/min. A small residue after thermal decomposition amounting to 5.75% of the initial sample weight resulted from a great susceptibility of AM-POSS to sublimation processes.

Chloroisobutyl POSS (HA-POSS) underwent a two-stage thermal decomposition. The first principal stage of thermal decomposition with a weight loss at Δ*T* between 220 and 420 °C, whose maximal rate at *T* = 325 °C amounts to 10.03%/min, was connected with the detachment of chloroalkyl group, whereas the second stage of thermal decomposition recorded at Δ*T* between 450 and 590 °C was connected with the degradation and decomposition of the cage structure. The residue after the thermal decomposition of HA-POSS amounted to 15.2%, which indicated its lower susceptibility to sublimation than that of AM-POSS.

Vinyl POSS (OL-POSS) underwent a four-stage thermal decomposition (Figure 5). The first three decomposition stages, whose maximal rate at *T*_1_ = 265 °C, *T*_2_ = 320 °C, and *T*_3_ = 375 °C amounted to 0.79, 1.80, and 0.36%/min, respectively, were connected with the degradation of unsaturated hydrocarbon chains and the detachment of hydrocarbon destruction products. The fourth stage of thermal decomposition was recorded at Δ*T* = 450–650 °C as in the case of HA-POSS, which was connected with the thermal degradation and decomposition of the cage structure. A very high residue after the thermal decomposition of OL-POSS, amounting to 86.5% at *T* = 600 °C, resulted from the susceptibility of this compound to cross-linking and thermal cyclization [27].

FTIR spectra of all studied POSS showed signals at wavenumbers of 745 and 837 cm^−1^, which were connected with stretching vibrations of the –CH group. A peak at wavenumber of 1091 cm^−1^ was connected with stretching vibrations of OH group, whereas a signal at wavenumber 1459 cm^−1^ corresponded to asymmetric stretching vibration of the –CH_3_ group. The peak at 2952 cm^−1^ was attributed to physically connected water at the POSS surface.

Additionally, the FTIR spectrum of POSS/AM showed signals at 1229 and 1570 cm^−1^, respectively. The first one was connected with valence vibrations, whereas the second was due to deformation vibrations of the –NH group.

**Figure 6:**
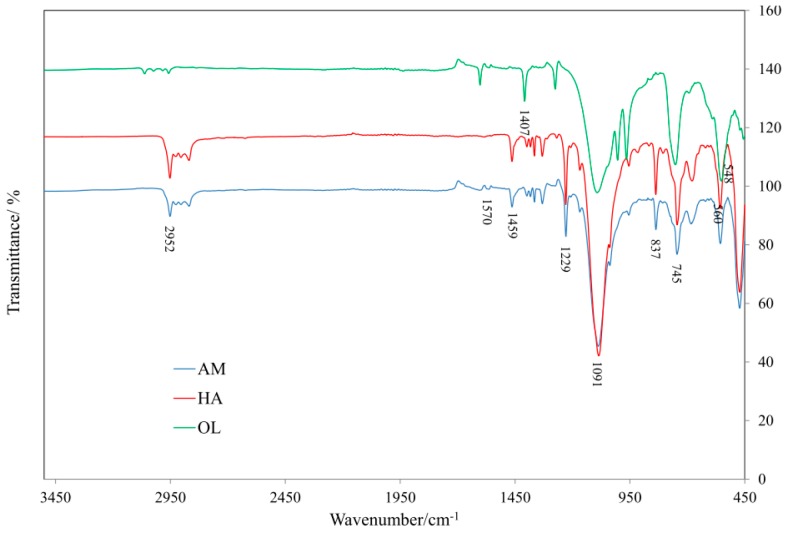
FTIR spectra of studied POSS.

IR spectra of POSS/HA showed a sharp signal at wavenumber 560 cm^−1^ that is connected with valence vibration of the C–Cl group. In the IR spectrum of the POSS/OL, a sharp peak at 548 cm^−1^ was observed, which was attributed to vibration of the CH=CH_2_ group, whereas the signal at 1407 cm^−1^ corresponded to deformation vibrations of the –CH group in –C=C–H (Figure 6).

### 3.3. IR Analysis of SR Composites

**Figure 7:**
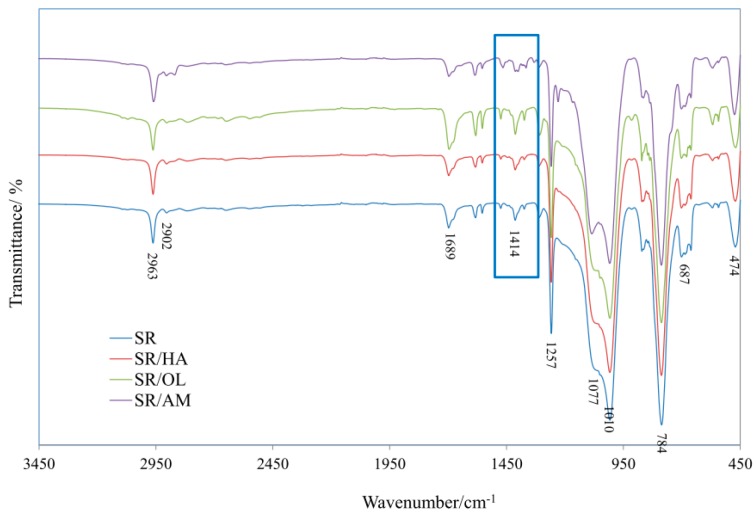
FTIR spectra of SR/POSS composites.

FTIR spectrum of silicone rubber showed sharp signals at wavenumbers 2963 and 2891 cm^−1^ that were connected with both the asymmetric and symmetric stretching vibrations of C–H group, respectively. Signals at wavenumbers 695, 1415, and 1258 cm^−1^ were connected with stretching vibrations of the C=C and C–H groups, and bending stretching derived from Si–CH_3_ groups, respectively [28]. Very strong signals at wavenumbers of 1077, 1010, and 787 cm^−1^ were connected with stretching vibrations of the Si–O–Si group (Figure 7).

The incorporation of silsequioxanes into the silicone rubber matrix did not significantly influence the character of the FTIR spectrum. Taking into account the increase in signal from 1410 to 1420 cm^−1^, in the case of SR/OL vulcanizate, one should state that the silsequioxanes investigated did not combine with silicone rubber chains during the process of reactive mixing and did not participate in the cross-linking of SR rubber.

### 3.4. Thermal Properties of SR Composites

The incorporation of polyhedral oligomeric silsequioxanes into the silicone rubber matrix decisively influenced the thermal properties of the rubber, expressed by the temperature of 5% and 50% of mass loss (*T*_5_ and *T*_50_), the thermal decomposition rate of the composite (*dm/dt*), the maximal temperature of its thermal decomposition (*T*_RMAX_), and the residue after its thermal decomposition (Pw) and residue at *T* = 650 °C (P_650_) (Table 3).

**Figure 8:**
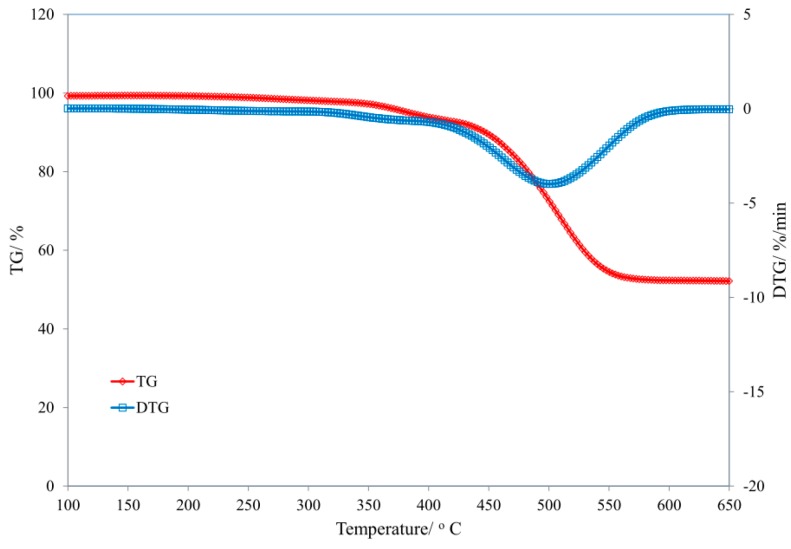
TG and DTG curves of SR/OL3 composite.

From the data listed in Table 3, it follows that all the silsequioxanes tested brought about an increase in the value of parameter *T*_5_. It should, however, be underlined that the highest increase in the value of this parameter was recorded for the composites containing OL-POSS. In the case of samples SR/OL3 and SR/OL6, parameter *T*_5_ was increased by 200 °C (sample SR/OL3) and by 194 °C (sample SR/OL6) in relation to the cross-linked SR rubber (Figure 8). These increases confirmed the susceptibility of vinyl silsequioxane to the processes of thermal cross-linking and cyclization.

An important thermal parameter, directly influencing the suppression of the flammability of elastomeric material, is its thermal decomposition rate (parameter *dm/dt*). The reduction in the destruction rate of polymeric composites exerts a positive influence on decreasing their flammability. This results from the formation of lower amounts of volatile and flammable products of pyrolysis passing to flame, which inhibits the free radical reactions proceeding in the combustion zone. Among all the samples tested, the lowest value of parameter *dm/dt* was shown by SR/OL3 and SR/OL6. These samples also showed the highest values of residue after thermal decomposition and at *T* = 650 °C (parameter Pw and P_650_), which distinctly indicated that vinyl groups of POSS/OL, non-active in the process of reactive mixing and rubber vulcanization carried out at *T* = 130 °C (Figure 5), took part in the reactions of cross-linking and cyclization proceeding during the thermal decomposition and combustion of SR rubber composites (Table 3).

The values of activation energy of thermal destruction of the SR rubber composites tested (Table 4) confirmed that POSS/OL, from among all the oligomeric silsequioxanes tested, catalyzed the processes of cross-linking and thermal cyclization to the highest extent.

The incorporation of melamine polyphosphate into the silicone rubber matrix, except for parameter *T*_5_, distinctly deteriorated its thermal stability. Under the influence of MPP, not only the thermal decomposition rate, *dm/dt*, of the composites increased, but also the residue after the thermal decomposition Pw and the residue at 650 °C decreased. Furthermore, melamine polyphosphate considerably reduced the cyclization of silicone rubber as confirmed by lower values of parameter Pw and P_650_ of SR/MPP composite in relation to the cross-linked SR (Table 5).

From the data listed in Table 5, it follows that composites containing synergic MPP system and silsequioxane were characterized by a higher thermal stability expressed by parameters *T*_5_, *dm/dt*, Pw, and P_650_ than the SR/MPP composite. A definite reduction in the thermal decomposition rate and an increase in the residue after thermal decomposition and at *T* = 650 °C of composite resulted from the formation of a ceramic surface layer that effectively blocked the transfer of mass and energy between the sample and flame.

The combustion of silicone rubber proceeds in three stages. The first stage is connected with its flameless thermal decomposition, accompanied by the emission of great amounts of gaseous destruction products. The next stage includes their ignition and a slow propagation of flame over the whole sample surface. The final stage of combustion is connected with the glow of combustion residue that assumes a form of a white uneven surface, under which is a brown heterogeneous layer.

The incorporation of polyhedral oligomeric silsequioxanes, POSS, into the silicone rubber matrix does not fundamentally change the mode of its combustion; nevertheless, it considerably reduces its flammability. From the test results listed in Table 6, it distinctly follows that the flammability of cross-linked SR rubber, especially in the presence of 6 parts by wt. of AM-POSS, HA-POSS, and OL-POSS was considerably reduced (Table 6).

**Figure 9:**
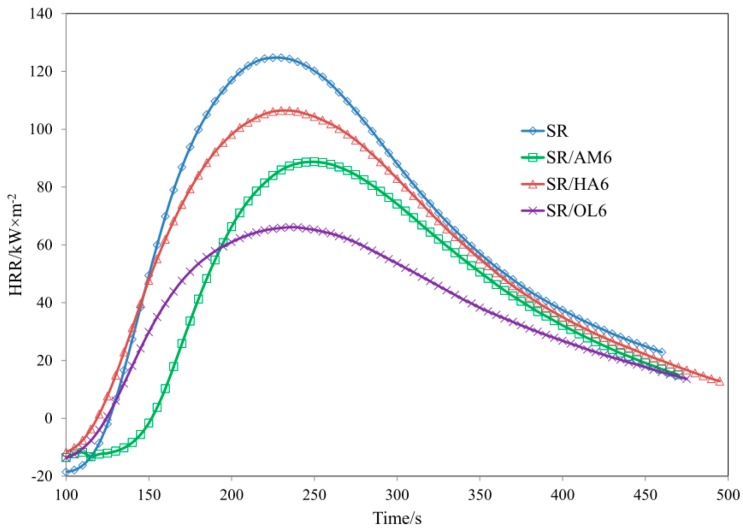
HRR curves of SR/POSS composites.

**Figure 10:**
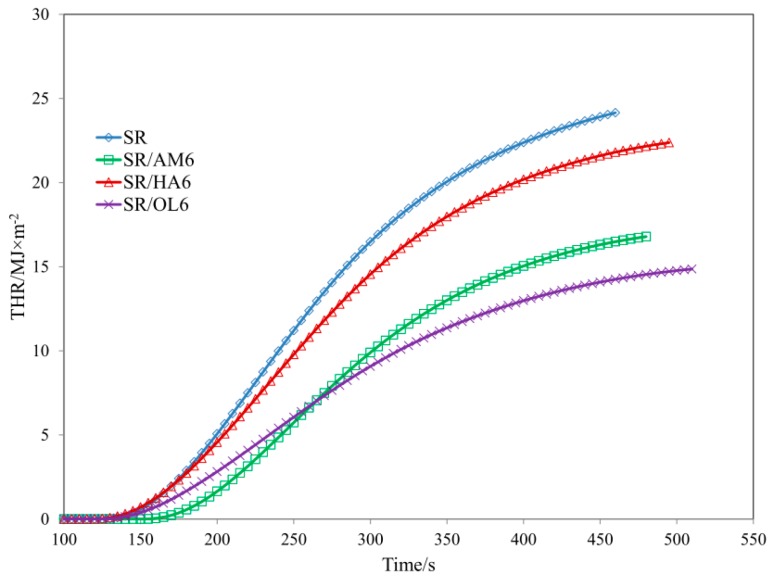
THR curves of SR/POSS composites.

In the presence of AM-POSS, the value the maximal heat release rate (HRR_MAX_) of SR/AM6 composite was decreased by 28.87% in relation to the cross-linked SR rubber, while it decreased in the case of HA-POSS by 14.65%, and in the case of OL-POSS by 47% (Table 6). Lower values of HRR_MAX_, but also those of the total heat release (THR), of POSS/SR composite, in relation to the unfilled, cross-linked SR rubber, were connected with the formation of a ceramic coating on the sample surface under combustion (Figure 9 and Figure 10).

It should be distinctly stressed that the silica formed during the thermo-oxidative decomposition of SR rubber, despite its high heat capacity, on account of low cohesion forces between its particles, does not lead to the formation of a homogeneous intumescent boundary layer that would efficiently reduce the effectiveness of fuel diffusion to flame and oxygen to the sample surface [1,29].

From the literature review and the test performed, it clearly follows that only in the presence of POSS, the boundary layer formed during the decomposition of silicone composites assumes a form of uniform ceramic coating that effectively reduces the mass and energy flow between sample and flame [11,23].

EDS analyses indicated that in the presence of POSS, the content of silicon increased with a simultaneous decrease in the oxygen content in the combustion residue of POSS/SR composites (Table 7).

The increase in silicon content confirmed the formation of ceramic structures suppressed in the boundary layer of burning POSS/SR composite, while the decrease in oxygen content in the combustion residue of the composite tested indicated the reduction in oxygen diffusion to the reaction zone and consequently the inhibition of thermo-oxidative processes by the ceramic layer formed during sample combustion.

The highest percentage silicon content with the lowest percentage content of oxygen was found in the combustion residue of SR/OL6 composite. The presence of unsaturated bonds in the cage structure of OL-POSS facilitated the cross-linking of residue after thermal decomposition and consequently the formation of a ceramic layer on its surface. The insulating character of the boundary layer of SR/OL6 composite was also confirmed by the parameter of mass loss rate (MLR), that in the case of the composite tested, was lower by more than 43% in relation to the cross-linked SR rubber (Table 6).

The effect on the SR/HA composite flammability is also exerted by the chlorine atom present in the HA-POSS structure. Halogens, passing to the gaseous phase of combustion, effectively interrupt the highly energetic radical reactions in both the pre- and flame-zones, consequently decreasing the efficiency of degradation and destruction processes of elastomeric composite [30,31].

The incorporation of melamine polyphosphate (MPP) to the silicone rubber matrix caused an increase in its flammability expressed by THR, HRR, and HRR_MAX_ parameters. The increase in the flammability of cross-linked silicone rubber in the presence of MPP resulted from two effects. First, the acidic products of the thermal decomposition of melamine polyphosphate incorporated into the silicone rubber matrix initiated the decomposition of the Si–O bond in the polymer main chain. Second, MPP, increasing the distances between polymeric chains, considerably reduced the thermal crosslinking process, especially the cyclization of SR rubber as confirmed by both the violent increase in the value of parameter MLR (Table 8) and the rate of thermal decomposition rate, *dm/dt*, with a simultaneous reduction in parameter *T*_50_ (Table 5). It should be distinctly underlined that the flame-retardant effectiveness of polyphosphates compounds reducing flammability directly depended on the availability of a carbon source, either in the form of an organic polymer or a carbonizing additive, such as pentaerythritol. The incorporation of MPP into the matrix of the poor in carbon SR rubber caused the acidic compounds, formed by the decomposition of melamine polyphosphate, to catalyze the decomposition of silicone rubber, practically decreasing the quantity of the homogeneous layer, insulating the carbon residue after the decomposition of SR rubber.

**Figure 11:**
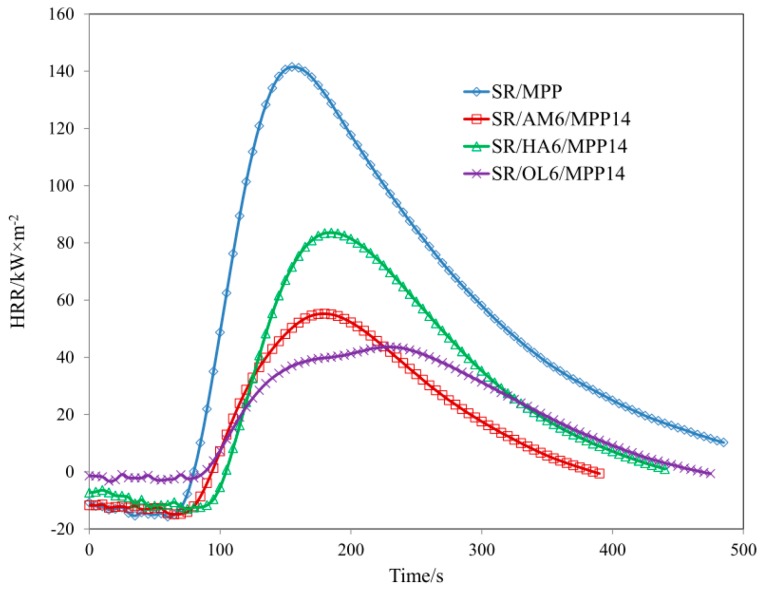
HRR curves composites of SR/POSS/MPP.

**Figure 12:**
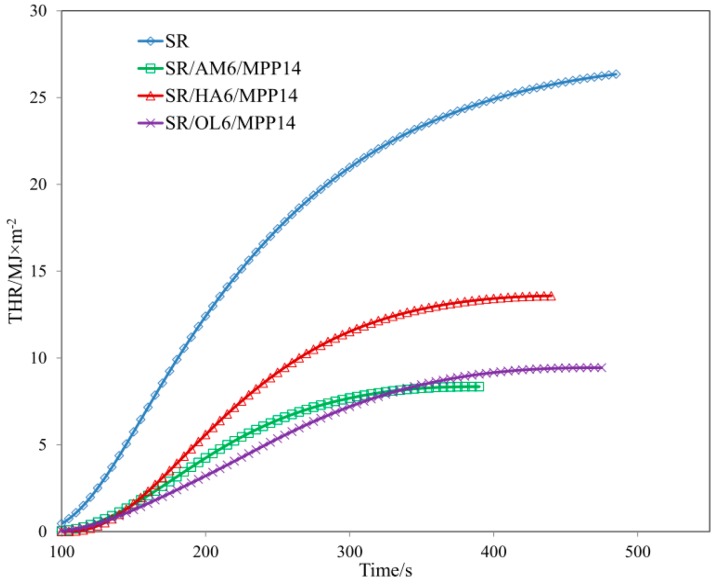
THR curves composites of SR/POSS/MPP.

From the data listed in Table 8, it follows that MPP in combination with POSS reduced the flammability of silicone rubber (Table 8, Figure 11 and Figure 12).

SR/AM6/MPP 14, SR/HA6/MPP14, and SR/OL6/MPP14 composites were characterized by lower values of parameter HRR_MAX_ by 60.83, 40.72, and 66.97%, respectively, in relation to the SR/MPP composite. Also, the parameters of mass loss rate (MLR) of SR/AM6/MPP 14, SR/HA6/MPP14, and SR/OL6/MPP14 underwent reduction by 17.5, 29.16, and 50.8%, respectively, in relation to that of the SR/MPP composite. The flame retardant action of the synergetic POSS/MPP system expressed by parameters HRR_MAX_, MLR, and THR (Figure 5, Table 8) and EHC (Table 8) resulted from the formation of a ceramic boundary layer that reduced the diffusion of oxygen to the boundary layer and decreased the intensity of degradation and thermal destruction processes of the composite, as well as constituted a barrier for the mass transport, and reduced the quantity of fuel passing to flame (Table 8) [32].

A lower flammability of SR/POSS/MPP composites in relation to that of SR/POSS resulted from the synergetic action of MPP and POSS during the formation of an insulating boundary layer during the thermal decomposition and combustion of SR/POSS/MPP composites.

The acidic destruction products of melamine polyphosphate initiate the degradation and thermal destruction processes of silicone rubber, which results in the formation of very high quantities of solid carbon products that pass to the flame zone, constituting an additional source of fuel (Parameter HRR_MAX_, MLR, SR/MPP composite) [33]. The ceramic structure formed in the presence of POSS, making it impossible to transfer carbon destruction products to flame, contributes to their cross-linking and cyclization, and consequently to increase the insulation capacity of the boundary layer formed during the decomposition and combustion of SR/POSS/MPP composites.

## 4. Conclusions

This paper presented flame retardant compounds for silicone rubber in the form of polyhedral oligomeric silsequioxanes (POSS), containing both isobutyl groups and amino-propyl (AM-POSS) or chloro-propyl group (HA-POSS) or vinyl groups (OL-POSS). Silsequioxanes were incorporated into the silicone rubber matrix in a quantity of 3 and 6 parts by wt. by the method of reactive stirring with the use of a laboratory mixing mill.

Based on the obtained results, it was found that the studied POSS, non-active in the process of reactive mixing and rubber vulcanization carried out at *T* = 130 °C, took part in the reactions of cross-linking and cyclization proceeding during the thermal decomposition and combustion of SR rubber composites.

Vinyl POSS, among all the polyhedral oligomeric silsequioxanes tested, catalyzed the processes of cross-linking and thermal cyclization of silicone rubber.

Based on the SEM-EDX analysis, it was found that the decrease of flammability of silicone rubber in the presence of POSS was connected with the formation, during thermal decomposition of POSS/SR composites, of a ceramic coating that limited flow mass and energy between sample and flame.

The increase in the flammability of cross-linked silicone rubber in the presence of MPP resulted from two effects. First, the acidic products of the thermal decomposition of melamine polyphosphate incorporated into the silicone rubber matrix initiated the decomposition of the Si–O bond in the polymer main chain. Second, MPP, increasing the distances between polymeric chains, considerably reduced the thermal crosslinking process, especially the cyclization of SR rubber.

A lower flammability of SR/POSS/MPP composites in relation to that of SR/POSS resulted from the synergetic action of MPP and POSS during the formation of an insulating boundary layer during the thermal decomposition and combustion of SR/POSS/MPP composites.

The acidic destruction products of melamine polyphosphate initiated the degradation and thermal destruction processes of silicone rubber, which resulted in the formation of very high quantities of solid carbon products passing to the flame zone, which constituted an additional source of fuel. The ceramic structure formed in the presence of POSS, making it impossible to transfer carbon destruction products to flame, contributed to their cross-linking and cyclization, and consequently to increasing the insulation capacity of the boundary layer formed during the decomposition and combustion of SR/POSS/MPP composites.

## Figures and Tables

**Table 1:** Chemical structure of POSS nanoparticles.

Material	Chemical Structure
AM-POSS	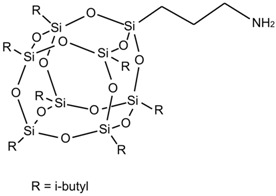
HA-POSS	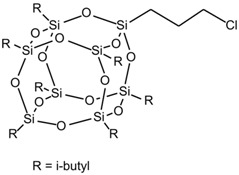
OL-POSS	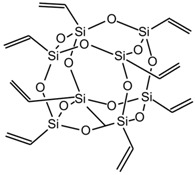

**Table 2:** Composition of the silicone (SR) rubber-based mixtures.

Sample Description	Component (phr)					
	SR	OP	POSS/AM	POSS/HA	POSS/OL	MPP
R OP	100	0.7	3	−	−	−
SR/AM3	100	0.7	−	−	−	−
SR/HA3	100	0.7	−	3	−	−
SR/OL3	100	0.7	−	−	3	−
SR/AM6	100	0.7	6	−	−	−
SR/HA6	100	0.7	−	6	−	−
SR/OL6	100	0.7	−	−	6	−
SR/MPP	100	0.7	−	−	−	20
SR/AM3/MPP17	100	0.7	3	−	−	17
SR/AM6/MPP14	100	0.7	6	−	−	14
SR/HA3/MPP17	100	0.7	−	3	−	17
SR/HA6/MPP14	100	0.7	−	6	−	14
SR/OL3/MPP17	100	0.7	−	−	3	17
SR/OL6/MPP14	100	0.7	−	−	6	14

SR—silicone rubber; OP—organic peroxide; POSS/AM—Aminopropylisobutyl polyhedral oligomeric silsequioxane; POSS/HA—Chloropropylisobutyl polyhedral oligomeric silsequioxane; POSS/OL—Vinyl polyhedral oligomeric silsequioxane; MPP—melamine polyphosphate.

**Table 3:** Thermal properties of SR/POSS composites.

Sample	*T*_5_ (°C)	*T*_50_ (°C)	*dm*/*dt* (%/min)	*T*_RMAX_ (°C)	Pw (%)	P_650_ (%)
SR	184	520	5.25	505	45.3	44.5
SR/AM3	320	−	4.74	520	49.0	49.5
SR/HA3	318	−	5.06	525	51.4	51.1
SR/OL3	384	−	4.87	506	51.3	50.2
SR/AM6	337	579	4.59	524	53.6	51.5
SR/HA6	304	−	5.40	517	52.5	52.3
SR/OL6	378	−	3.91	514	52.3	52.4

*T*_5_, *T*_50_—temperature of sample 5% and 50% mass loss, respectively; *dm/dt*—maximum rate of thermal decomposition of vulcanizates; *T*_RMAX_—temperature of maximum rate of thermal decomposition of vulcanizates; Pw—residue after the thermal decomposition of vulcanizates; P_650_—residue after heating to *T* = 650 °C.

**Table 4:** Activation energy values of SR/POSS composites.

Sample	Ea (kJ/mol)
SR	46.57
SR/AM6	51.84
SR/HA6	84.17
SR/OL6	98.22

**Table 5:** Thermal properties of SR/POSS/MPP composites.

Sample	*T*_5_ (°C)	*T*_50_ (°C)	*dm/dt* (%/min)	T_RMAX_ (°C)	Pw (%)	P_650_ (%)
SR	184	520	5.25	505	45.3	44.5
SR/MPP	315	420	17.65	408	32.4	28.1
SR/AM3/MPP17	313	424	13.32	410	32.8	29.2
SR/AM6/MPP14	305	435	10.71	415	35.1	32.9
SR/HA3/MPP17	380	418	16.16	412	33.4	30.7
SR/HA6/MPP14	328	435	10.08	415	36.2	33.9
SR/OL3/MPP17	340	421	14.81	412	33.6	34.2
SR/OL6/MPP14	378	440	9.89	415	35.6	36.1

**Table 6:** Flammability of SR/POSS composites.

Sample	TTI (s) ± 5	THR (MJ/m^2^) ± 1	HRR (Kw/m^2^) ± 2	HRR_MAX_ (kW/m^2^) ± 2	EHC (MJ/kg) ± 2	EHC_MAX_ (MJ/kg) ± 1	MLR (g·m^−2^·s^−1^) ± 0.01
SR	93	21.6	73.62	124.73	37.22	79.82	0.064
SR/AM3	91	18.1	59.81	102.67	38.88	77.80	0.049
SR/HA3	80	20.3	73.59	120.26	36.50	61.50	0.051
SR/OL3	85	19.3	62.49	105.50	34.41	76.49	0.061
SR/AM6	114	14.1	52.81	88.72	30.49	61.44	0.057
SR/HA6	106	20.7	60.58	106.45	38.1	77.42	0.050
SR/OL6	99	13.5	38.72	66.10	31.72	78.05	0.036

TTI—time to ignition; THR—total heat release; HRR—heat release rate; HRR_MAX_—maximal heat release rate; EHC—effective combustion heat; EHC_MAX_—maximal effective combustion heat; MLR—mass loss rate.

**Table 7:** EDS results residue after burning.

Sample	C%	Si%	O%	P%
SR	−^1^	51.60	48.40	−
SR/AM6	−^1^	53.87	46.13	−
SR/HA6	−^1^	54.96	45.04	−
SR/OL6	−^1^	55.64	44.36	−
SR/MPP	10.00	38.27	49.94	1.79
SR/AM6/MPP14	9.59	40.90	48.40	1.11
SR/HA6/MPP14	4.48	42.45	51.01	2.06
SR/OL6/MPP14	3.00	45.36	49.37	2.27

^1^ small amount of carbon.

**Table 8:** Flammability composites of SR/POSS/MPP.

Sample	TTI (s)	THR (MJ/m^2^) ± 1	HRR (Kw/m^2^) ± 2	HRR _MAX_ (kW/m^2^) ± 2	EHC (MJ/kg) ± 2	EHC _MAX_ (MJ/kg) ± 1	MLR (g·m^−2^·s^−1^) ± 0.01
SR/MPP	56	24.5	74.04	141.48	27.95	79.13	0.120
SR/AM3/MPP17	56	14.2	42.18	78.25	29.49	60.33	0.052
SR/AM6/MPP14	63	7.9	30.47	55.22	13.61	59.06	0.099
SR/HA3/MPP17	54	17.4	69.75	114.42	25.31	77.38	0.092
SR/HA6/MPP14	83	12.3	49.95	83.58	22.46	75.77	0.085
SR/OL3/MPP17	70	11.5	29.26	50.12	21.15	73.54	0.049
SR/OL6/MPP14	72	9.2	27.52	43.63	14.24	75.85	0.059
